# Current Status of the Evaluation and Management of Lupus Patients and Future Prospects

**DOI:** 10.3389/fmed.2021.682544

**Published:** 2021-05-28

**Authors:** Sule Yavuz, Peter E. Lipsky

**Affiliations:** ^1^Department of Medical Sciences, Rheumatology, Uppsala University, Uppsala, Sweden; ^2^Ampel BioSolutions and Re-Imagine Lupus Investigation, Treatment and Education Research Institute, Charlottesville, VA, United States

**Keywords:** systemic lupus erythematosus, lupus nephritis, transcriptomic (RNA-Seq), lupus genetics, lupus treatments

## Abstract

The vastly diverse nature of systemic lupus erythematosus (SLE) poses great challenges to clinicians and patients, as well as to research and drug development efforts. Precise management of lupus patients would be advanced by the ability to identify specific abnormalities operative in individual patients at the time of encounter with the clinician. Advances in new technologies and bioinformatics have greatly improved the understanding of the pathophysiology of SLE. Recent research has focused on the discovery and classification of sensitive and specific markers that could aid early accurate diagnosis, better monitoring of disease and identification of appropriate therapy choices based on specific dysregulated molecular pathways. Here, we summarize some of the advances and discuss the challenges in moving toward precise patient-centric management modalities in SLE.

## Key Points

- Substantial diversity in clinical pictures of SLE is likely the reflection of the underlying molecular heterogeneity.
- Measuring disease activity and predicting a flare timely in SLE need instruments that are reliable, practical, and sensitive to capture change between mild and moderate DA.
- Integrating advances in new technologies and bioinformatics to clinics may yield more clinically useful tools for better patient care and innovative trial designs.
- Patient-centric approach aims at individualized profiling of each patient to assess the main dysregulated pathway (s) and offer the best adequate treatment by predicting the risk of damage in SLE.

## Introduction

Systemic lupus erythematosus (SLE) is a very challenging disease for physicians. Given the considerable heterogeneity between individuals as well as within the same individual over time, several aspects of lupus remain uncertain and create substantial unmet medical need. Some of these include early diagnosis, accurate measurement of disease activity, prediction of disease course and flares and tailoring treatment based on the specific patient's need.

Research, improvements in patient care, and the use of immunosuppressives have changed the course of SLE. Survival has improved dramatically over the past 5 decades from a 5-year survival of 50% to a 15-year survival of 85% with current medical regimens (1, 2). Despite expanding knowledge, further improvement in survival has mostly stalled, and the rate of progression to renal failure has not improved. Mortality still remains approximately double that of the age-matched general population, with the greatest increase in relative risk in the youngest patients (3–5). Notably, the chance of being dead at the age of 35 for a patient diagnosed with SLE at the age of 20 is one in seven. Moreover, the morbidity linked to the disease itself or side effects of medications, especially damage related to long-term use of glucocorticoids, remains an unresolved problem (6, 7). The outlook for patients with lupus nephritis has not dramatically changed over the last years, as 10% of patients still progress to end-stage renal disease (8).

The link between disease activity, damage, and mortality has been established (9, 10). However, the reproducibility of disease activity is less well-documented, and the use of established instruments is not commonplace in clinical practice. Measuring disease activity accurately and in a timely manner is essential not only for better patient care but also for the success of clinical trials. However, currently available instruments are problematic. An optimal tool needs to be reliable, practical, and sensitive to capture change longitudinally. Importantly, for widespread adoption, it must be logical to a health care professional (HCP), incorporate elements of patient concerns, and impose a minimal administrative burden. Additional challenges arise when considering well-recognized ethnic disparities, with the highest SLE disease burden in non-white populations. Observed disparities also exist at the genetic and cellular level (11–14); patient ancestry impacts gene expression patterns significantly in SLE as well as the dominant molecular pathways active in disease pathogenesis and response to therapeutics also appears to be affected (15, 16). However, the majority of clinical trials consist disproportionately of more European Ancestry (Caucasian) patients (17). Moreover, in clinical practice, ancestral diversity is rarely considered when treatments are prescribed, even though there is considerable evidence of differential responsiveness to both standard of care and off-label medications (18, 19).

Clinical trials have also been affected by the complexity of characterizing SLE patients precisely. The results of clinical trials in SLE have been largely disappointing compared to successes in rheumatoid arthritis, psoriatic arthritis and spondylitis. Only two agents, Belimumab, a monoclonal antibody that binds BAFF (TNFSF13B), and very recently, Voclosporin have been approved for SLE and LN, respectively, since 1959 (16, 20). The possible reasons are discussed in detail elsewhere (21), but a hint may be hidden in the trials showing that up to 30% of established SLE patients screened for a new therapy are anti-nuclear antibody (ANA) negative. This observation is surprising because ANA was previously thought to be persistently present in most if not all SLE patients (22, 23), and spurs several questions around ANA, such as the possibility of ANA positive SLE patients (so called immune-active) may respond to some therapies differently from ANA negative patients. This possibility raises the interesting question as to whether ANA positivity might be a theragnostic in SLE (24).

Recently, several immunologic discoveries and genetic association studies in SLE have immensely improved understanding of the pathophysiological mechanisms underlying SLE molecular and clinical heterogeneity. Defining the molecular traits and developing innovative tools that integrate molecular pathways and clinic manifestations may transform current clinical approaches to SLE and provide the basis for patient-centric precision medicine in SLE.

## Early Diagnosis

Diagnostic delays and misdiagnosis are common in SLE. A survey of more than 2,500 UK lupus patients in 2014 showed that the mean time between patients' first awareness of SLE symptoms and actual diagnosis was 6.4 years and half of the patients reported that they had been misdiagnosed initially (25). Diagnosis in SLE relies on the physician's clinical judgment based on a combination of clinical signs/symptoms and available clinical tests (most frequently ANAs). Early diagnosis and effective treatment may prevent long-term complications that cause increased morbidity and mortality. Given the considerable heterogeneity in most disorders in rheumatology, including SLE, the development and validation of diagnostic criteria can be quite challenging. As a result, there are only a few validated diagnostic criteria in rheumatology (26). Most efforts have been directed toward developing more precise classification criteria that aim to assemble cohorts that are representative of the majority with disease for clinical research. In practice, on the other hand, physicians often use classification criteria to diagnose complex diseases like SLE (27). Ideally, when the sensitivity and specificity ~100%, it is possible to employ the terms interchangeably, although this is not recommended. Whereas, classification criteria tend to include features phenotypically more prevalent in relevant diseases, the use of classification criteria tends to leave a group of patients with an incomplete set of criteria or uncommon features misclassified. The development process of recent ACR/EULAR SLE classification criteria (28–30) took into consideration these issues, such as applicability to early or new onset lupus without compromising specificity and focusing on true autoimmune disease. In this context, the 2019 EULAR/ACR criteria performed well in early disease, defined as 1 to <3 years of disease duration, with a sensitivity better than the former ACR criteria (97 vs. 81%) and a specificity better than the 2012 SLICC criteria (96 vs. 88%) (31).

Data has shown that autoantibodies, such as ANA, appear in the blood as early as 9.4 years (mean 3.3 years) before the clinical onset of SLE and the pattern evolves, reflective of the disease process. Closer to the onset of overt disease, more specific autoantibodies (e.g., anti-dsDNA, anti-Sm) are detected (32). Given the low specificity of ANA for SLE, screening patients with non-specific symptoms becomes a burden for many rheumatology clinics across the globe, as it is positive in up to 20% of individuals without SLE. This is also the rationale behind the 200-antigen immune-chip assay, SLE-key (ImmunArray, Richmond, VA, USA), which claimed a sensitivity of 94% in ruling out the non-diseased subjects, but is no longer available. Another assay detecting cell-bound complement activation products *(Exagen, Vista, CA, USA)*, which outperforms anti-dsDNA by up to 48%, has been in use for assisting physician in diagnosing SLE for some time (33).

SLE is heavily influenced by genetics, and so far, ~100 genetic susceptibility loci have been identified (14). Recent advances have prompted the possibility of utilizing genetic risk scores (GRS) as tools for predicting disease susceptibility and outcome in SLE (34, 35). GRS are numeric scores that combine a large number of disease-associated genetic variants that are weighted by SLE risk odds ratios and reflect the disease associated genetic load in an individual patient (36). Knevel et al. (37) have developed a GRS (G-PROB) using genome-wide significant variants (*p* ≤ 5 × 10^−8^) from previously published genome-wide association studies (GWAS), and tested its potential as a diagnostic tool in a set of patients with inflammatory arthritis (rheumatoid arthritis, systemic lupus erythematosus, spondyloarthropathy, psoriatic arthritis, and gout). Coupled with good discriminatory capacity (area under the curve, AUC: 0.69–0.84), it could single out a likely diagnosis for 45% of patients with a positive predictive value of 0.64, that could be further improved with serologic data. Despite only being tested in Caucasian cohorts, the results of this study demonstrate the potential clinical utility of the GRS for diagnosis, especially when incorporating serologic findings, such as ANA, and, possibly, clinical manifestations.

Transcriptomic analysis might also contribute to diagnosis. Based on a meta-analysis of 40 independent publicly available gene-expression studies containing 7,471 transcriptomic profiles, Haynes et al. identified a core gene set (93-gene signature, SLE MetaSignature) that is dysregulated in patients with SLE and distinguishes SLE from other relevant rheumatic disorder and infections (38). They further validated the SLE MetaSignature in a prospective study comprising patients with juvenile onset SLE, juvenile idiopathic arthritis, and healthy subjects. This study demonstrates the potential value of the integration of gene-expression studies into the clinic as a means to improve the diagnosis of SLE.

## Challenges in Established Disease

### Disease Activity

Loss of tolerance to nucleic acids and sustained autoantibody production along with an imbalance between the production of apoptotic material and its disposal are the key features in SLE pathogenesis. In addition to the complex genetic background in the majority of the patients, epigenetic, and environmental factors may contribute to the disease and influence the occurrence of flares. The diverse clinical picture of SLE likely reflects underlying molecular heterogeneity coupled with unpredictable fluctuations in disease activity perhaps reflective of environmental triggers, which leads to organ damage accrual, challenges clinicians, and hampers clinical trials. Different instruments have been developed to measure general disease activity, of which the Systemic Lupus Erythematosus Disease Activity Index (SLEDAI) and the British Isles Lupus Assessment Group (BILAG) are predominantly used in clinical trials (39). However, none of these instruments is perfect and neither is routinely used in clinical practice (Table 1). Traditional biomarkers, such as complement and anti-dsDNA, are commonly used to detect a flare; however, fluctuations in serology may not predict a flare as shown in a long term follow-up of a cohort of serologically active but clinically quiescent patients (40). Moreover, these tests have been studied most in patients with lupus nephritis and their utility in detecting other manifestations of SLE is less clear. More comprehensive tools that integrate novel biomarkers, identified by longitudinal analyses of well-characterized cohorts and the use of new big data analytical techniques, such as machine learning, could be valuable for capturing disease activity and providing timely stratification of patients based on the unified pattern of dysregulated pathways (41, 42). Transcriptomic studies have identified distinctive signatures in SLE blood samples that include type I interferons (IFN), as well as myeloid and B cell-related (plasma cell) signatures (43–46). Several studies also showed a correlation between these signatures and disease activity (42–44). Although a robust interferon gene signature (IGS) has been demonstrated in both blood and affected tissues of SLE patients (47), no apparent correlations have been found between the IGS and disease activity in longitudinal studies (46, 48, 49). Both type I and type II IFNs (IFNγ) contribute to the IGS. However, only modest differences in enrichment of genes downstream of specific IFNs in cells and tissue have been demonstrated in SLE (46). The aforementioned study also uncovered that the prolonged IGS in monocytes, even when disease activity is low, is likely the reason that the IGS is not useful in the detection of disease activity accurately.

**Table 1:** Some of the potential urinary biomarkers.

	**Diagnostic**	**Prognostic**	**Correlation with renal histology**
*Cytokine*			
TWEAK	**+**	**+**	
IL-17	**+**	**+**	**+** (proliferative LN)
BAFF	**+**	**+**	
TGF-β	**+**		**+** (proliferative LN)
sCD163		**+**	**+** (inflammation)
*Chemokines*			
CCL2/MCP-1	**+**	**+**	
CXCL10/IP10	**+**	**+**	**+** (proliferative LN)
CXCL4	**+**		**+** (proliferative LN)
*Vascular molecules*			**+** (Class IV LN)
VCAM	**+**		
Angiostatin	**+**		
ALCAM	**+**		
*Urinary enzyme*			
NGAL	**+**	**+**	

*TWEAK, TNF-like weak inducer of apoptosis; BAFF, B cell activating factor; TGF-β, transforming growth factor beta; CCL2, chemokine (C-C motif) ligand 2/MCP-1, monocyte chemoattractant protein-1; CXCL10, C-X-C motif chemokine 10/IP-10, Interferon gamma-induced protein 10; CXCL4, C-X-C motif chemokine 4; VCAM-1, vascular cell adhesion molecule 1; ALCAM, activated leucocyte cell adhesion molecule; NGAL, neutrophil gelatinase-associated lipocallin*.

Epigenetic mechanisms have also emerged as important factors in SLE pathogenesis (48, 49). Aberrant changes in DNA methylation, histone modification, and non-coding RNAs, induced by environmental influences or regulated by genetic factors, can alter gene expression. Epigenetic modifications may vary depending on the location, cell type, ethnicity, response to therapy, and the course of the disease. Studies of DNA methylation, which is the most widely studied epigenetic marker, have consistently shown demethylation at interferon-regulated genes across all the investigated cell-types in SLE, independent of disease activity (50, 51). Notably, altered DNA methylation of particular genes involved in T cell biology and genes regulated by interferon seems to be associated with lupus disease activity and show promise as markers for disease monitoring (52, 53). So far, most epigenetic studies in lupus have been cross-sectional, and epigenetic changes over time and their relation with disease activity have been unclear. In this respect, a recent longitudinal study assessing the DNA methylome of neutrophils across ancestries has reported two differentially methylated CpG sites unique to African-American patients that are associated with lupus disease activity (54). MicroRNAs (miR), small non-coding RNA molecules, are closely linked to DNA methylation and thus to epigenetic changes that regulate the expression of multiple genes. Serum miR levels change during active or inactive disease states and may serve as a disease biomarker. In fact, miR-146, miR-21, and miR-148a correlate with lupus disease activity and have been proposed as biomarkers (55, 56).

### Lupus Nephritis and Application of Cutting Edge Technologies

Lupus nephritis (LN) is a common feature of SLE that causes higher morbidity and mortality (8, 57). As LN is usually asymptomatic, the common practice is the regular screening of urine samples of SLE patients for proteinuria or active sediment. When either of these exists, a renal biopsy, as the “gold standard,” is frequently employed to confirm diagnosis and guide treatment. However, the current histopathological classification system has some limitations. It focuses mainly on glomerular lesions and interstitial lesions are incompletely addressed. This is a deficit because recent evidence suggests that interstitial involvement is predictive of progression to renal failure (58).More importantly, the underlying molecular pathways cannot be assessed by routine histology or immunofluorescence studies or even electron microscopy. It should be emphasized that only 30% of LN patients achieve a complete remission with the current treatment paradigm.

Leveraging powerful technologies, such as single-cell transcriptomics, could provide new insights into the diverse mechanisms involved in the pathogenesis of tissue injury. Information from these studies may advance current understanding of the complex interaction between immune and resident cells in the kidney as well as identify drivers of renal flares; the result could be novel biomarkers as well as the development of clinically meaningful user-friendly metrics for prognosis and treatment response. In this context, single-cell transcriptomic analyses show different clusters of myeloid, T, and B cell subtypes, where NK and CD8^+^ cytotoxic T cells are major proliferating populations in LN kidney samples (59). By contrast, few B cells are detected in healthy kidneys by either flow cytometry or single-cell mRNA sequencing, whereas resident macrophages and memory CD4^+^ T cells are the dominating cell populations. Neutrophil signatures in the blood are also associated with active LN (43, 60), whereas single-cell transcriptomic profiles of neutrophils have not yet been identified in the LN kidney. Kidney epithelial cells also contribute to disease progression in LN, as demonstrated by profiling unselected kidney cells in a parallel study. Tubular epithelial cells differentially express the IGS and fibrosis-associated genes are found in those samples from patients who had an inadequate response to treatment (61). Despite these interesting results, it is still unclear how different disease states, background treatment, and ancestry influence the composition of cells and their gene expression. Although there are well-recognized limitations of single-cell RNA sequencing, these initial studies provide a framework for understanding the immune cell types potentially contributing to LN (62, 63). Questions that are more specific, such as whether T and B cells in the kidney are antigen-specific or spatial relationships among detected cells should also be addressed in future studies.

Changes in lupus kidneys are dynamic, and longitudinal tissue sampling would facilitate tracking disease progression and response to the intervention. However, renal biopsy is not entirely risk-free. Conventionally used biomarkers such as proteinuria or serologic markers have a limited ability to predict renal prognosis adequately, and persistent proteinuria can be secondary to residual activity or chronic damage. Given that the urine is easily accessible and urinary biomarkers may directly reflect the underlying disease process in the lupus kidney, several candidates have been identified (Table 2). The repertoire of potential urinary biomarkers has been somewhat disappointing, however, as only a limited number has been validated in longitudinal cohorts, few have been tested in multiple ancestries and none has been successfully used in clinical trials (64). The sources of proteins in the urine are both from circulation and local cells (infiltrating immune or resident) in the kidney, thus potentially rendering urine a valuable source of biomarkers for both systemic and intrinsic kidney diseases. Advances in proteomic techniques allow for screening a large number of proteins simultaneously with the aim of discovering novel biomarkers. A recent high throughput urine proteome study integrated with single-cell transcriptomic of lupus kidneys detected a chemokine profile, inducible by IFN-γ, which is produced mainly by CD8^+^ T cells in active LN patients (65). Although this study was limited by the sample size, it is an encouraging example of the potential of urine proteome profiling to predict active immune pathways in the kidney.

**Table 2:** Comparison of two commonly used instruments in clinical trials.

**Instrument**	**Content**	**Validity evidence**	**Ability to detect change**	**Training needed**
BILAG 2004	DA within last 1 month	Yes	Yes	Yes
SLEDAI-2K	DA within last 1 month	Yes	No improvement or worsening in organ systems	No

*BILAG 2004, updated version of British Isles Lupus Assessment Group; Systemic Lupus Erythematosus*.

*Disease Activity Index 2000; DA, Disease activity*.

### Outcome Measures

Given the link among disease activity, damage, and mortality, targeting low disease activity (LDA) seems a logical treatment goal in SLE, in which remission, especially durable drug free remission, is difficult to achieve (66) (Box 1). The Asia Pacific Lupus Collaboration has generated the Lupus Low Disease Activity State (LLDAS) metric for generalized SLE, which has shown to be associated with reduced damage accrual (67–69). Retrospective studies suggest that LLDAS might have value as a outcome measure in clinical trials (69, 70). The Lupus Multivariable Outcome Score (LuMOS), a multivariable response score, was developed to address the inherent heterogeneity of SLE patients, which may be responsible for the frequent failure of the responder indices used in clinical trials. LuMOS utilized information collected from different variables using the Study of Belimumab in Subjects with SLE 76-week (BLISS-76) as training dataset and an independent dataset from the BLISS-52 trial as validation (71).The LuMOS outperformed SRI-4, the original outcome measure, in the BLISS-52 trial (72) in terms of the effect size. However, data from the RCTs in SLE other than belimumab and different subpopulations or longitudinal studies in well-defined SLE cohorts may be necessary to provide further justification for its performance in comparison to other outcome measures used in clinical trials in SLE.

Box 1 **Lupus low disease activity state (65).**

**Disease activity**

- SLEDAI-2K ≤ 4, with no activity in major organ systems (renal, CNS, cardiopulmonary, vasculitis, fever) and no hemolytic anemia or gastrointestinal activity
- No new features of lupus disease activity compared with the previous assessment
- SELENA-SLEDAI physician global assessment (PGA, scale 0–3) ≤ 1

**Immunosuppressive medications**

- Current prednisolone (or equivalnt) dose ≤ 7.5 mg daily
- Well-tolerated standard maintenance doses of immunosuppressive drugs and approved biological agents, excluding investigational drugs SLEDAI-2K, Systemic Lupus Erythematosus Disease Activity Index 2000.

SELENA-SLEDAI, Safety of Estrogen in Lupus Erythematosus National Assessment-Systemic Lupus Erythematosus Disease Activity Index. PGA, Physician Global Assessment

Organ-specific disease features and their contribution to end-organ damage have been well-described for the kidney, skin, and central nervous system (CNS) (73). Given the different weighing of clinical manifestations in different outcome measures, two patients with entirely different clinical features may yield the same score. As considerable clinical diversity exists, a significant response detected with one of these tools might not necessarily reflect a meaningful clinical response for clinicians and patients in SLE. Even within the same organ system, for instance, skin involvement, lupus rash may exhibit a different degree of activity between two patients. If one has 5% of the body surface (skin color: faint erythema) covered, whereas the other has 15% (skin color: dark red), nevertheless, both of them get the weighted score of 2 on SLEDAI-2K, rash as a descriptor. For individualized treatment, disease activity and organ involvement need to be assessed objectively and accurately. Should that be the case, it would be essential to ascertain the specific goal and the outcome measure (general SLE, organ-specific, or both) that is most appropriate. To this end, the academic community has developed the Cutaneous Lupus Area and Severity Index (CLASI) to assess skin activity and capture differences, but this has not yet been accepted by regulatory authorities as a validated outcome measure in clinical trials (74).

As stated above, predicting disease flare accurately and preventing it is an essential task for clinicians as recurrent flares are associated with organ damage accrual and mortality in SLE (9, 75). Given its obvious relevance to optimal patient care, measuring flare as an endpoint in clinical trials should also be considered with a physician-friendly carefully devised instrument that could discriminate moderate to mild flares reproducibly (41).

### Patient Satisfaction and Fatigue

Data show that a patient's active participation in her/his healthcare decisions with physicians may improve outcomes and lead to higher patient adherence and satisfaction (76, 77). However, there are often gaps between patient's preferences and what physicians think is the best for patient care. Physicians usually take into account a range of clinical and laboratory outcomes to consider SLE treatment satisfactory, whereas patients mostly prioritize manifestations that impact their daily functioning. Fatigue is one of the most common (70–90%) features of SLE and can be both debilitating and difficult to treat, and clearly, represents a major unmet need with substantial impact on the patient's daily functioning and quality of life (78–80). Both the FDA and the European Medicines Agency (EMA) highlight fatigue as being an important patient-reported outcome (PRO). FDA advises using PROs as secondary outcomes in SLE clinical trials; however, no specific instrument for measuring the level of fatigue has been recommended.

The causes of fatigue in lupus are poorly understood and several possible mechanisms are likely to contribute (Table 3). Fatigue is a common (80–90%) dose-dependent side effect of interferon (IFN)-α treatment of HCV or malignancy (81), and type 1 IFNs are known to play a central role in SLE pathogenesis. As circulating IFN-α may not easily cross the brain blood barrier, it may exert its central effect *via* other cytokines that are induced in SLE, such as TNF, IL-1, and IL-6, all of which are implicated in the pathogenesis of chronic fatigue as well (81, 82). However, attribution of fatigue to an immunologic process directly associated with immune alterations in SLE has been extremely difficult. This may be related to underpowered studies or the lack of sufficient tools to capture changes in fatigue levels. In a recent study comprising 426 SLE patients, a close correlation was found between circulating antibodies to the NR2 subunit of the N-methyl-D-aspartate receptor (NMDAR) and the severity of fatigue in addition to the clinical disease activity index (Systemic Lupus Erythematosus Disease Activity Index 2000) and anti-double stranded DNA antibodies, independently of the presence of neuropsychiatric lupus manifestations (83). The presence of anti-NR2 antibodies may be a helpful diagnostic tool in the evaluation and therapeutic management of fatigue. The BLISS trials investigating the use of Belimumab in SLE showed improvement of fatigue scores measured by the FACIT-fatigue (FACIT-F) scale (84). Interestingly, using belimumab for at least 6 months affected the levels of anti-NR2 antibodies along with fatigue severity (34).

**Table 3:** Possible contributors of fatigue.

• Metabolic causes
° Hypothyroidism, anemia, vitamin D deficiency
• Medication
° Glucocorticoids, immunosuppressives
• Sleep disturbance and fibromyalgia
° Pain, depression, or anxiety
• Central nervous system involvement
• Inflammation related
° Abnormal oxidative metabolism and mitochondrial dysfunction

On the other hand, fatigue could also be a result of corticosteroid withdrawal. In a recent RCT comparing two different administrations of the same daily dose of prednisone, the delayed-release in the evening compared to the immediate-release in the morning in patients previously receiving the same dose of corticosteroids, a significant improvement in fatigue levels was noted in both arms, compared to that present at entry. This may imply that one component of fatigue may be linked to the difference between the actual administered dose of prednisone by the patient and what is being prescribed (85).

## Toward Patient-Centric Clinical Trials in SLE

The introduction of biological and targeted disease-modifying drugs into clinical practice has revolutionized the outlook of patients with RA, psoriatic arthritis, and ankylosing spondylitis. Unfortunately, SLE lags far behind these achievements in other inflammatory arthropathies, and belimumab is the only *biologic* approved for the treatment of SLE and LN in the last 60 years (86). Failure to achieve primary end-points of many study drugs in different clinical trials in SLE has led to several treatment guides that contain recommendations based on clinical experience or eminence-based rather than evidence-based ones for physicians (87–89). The lack of biomarkers to forecast treatment response to individual drugs limits the ability to prevent patients from cycling through a few approved and unapproved drugs and protect them from unnecessary side effects of immunosuppressive drugs or glucocorticoids. So far, a considerable amount of valuable information has been gleaned from *post-hoc* analyses of these failed trials. Of note, however, because of substantial molecular heterogeneity in SLE, inclusion of patients with molecular subtypes lacking the target of the drug imposes problems when interpreting the trial results. Some of the issues that might lead to the failure of clinical trials in SLE are listed in Box 2.

Box 2 **Some of the problems in SLE drug development.**

- Disease heterogeneity
  ° molecular heterogeneity
- Trial size
- Selection bias in patient population (Ancestry/ethnicity)
  ° African, Asian, Hispanic, and Aboriginal ancestry develop SLE earlier and tend to have higher disease activity and mortality, yet underrepresented in trials
- Endpoint definitions
- Trial duration
  ° some endpoints can be achieved beyond 52 weeks
- Background therapy
- Lack of reliable biomarker
- Patient for the trial vs. trial for the patient?

More recently, the second of 3 trials of anifrolumab (TULIP-2), an antibody against the type-I interferon receptor, has shown a significant effect on the primary end point of response, according to British Isles Lupus Assessment Group (BILAG) based composite lupus assessment (BICLA) at week 52 (90). TULIP-2 has also pointed out the challenges that exist in the trial design of SLE for the selection of endpoints, as the TULIP-1 trial did not reach significance for its primary endpoint, the Systemic Lupus Erythematosus Responder Index (SRI-4) (72, 91). Both of these instruments were devised during the development of the belimumab and epratuzumab trials, and were expected to provide concordant results (92). Even these two almost identically designed TULIP trials with conflicting results underline that clinical trials may be benefited from the re-evaluation of the outcome measures that define more clinically relevant endpoints and their integration into trials.

**Figure 1:**
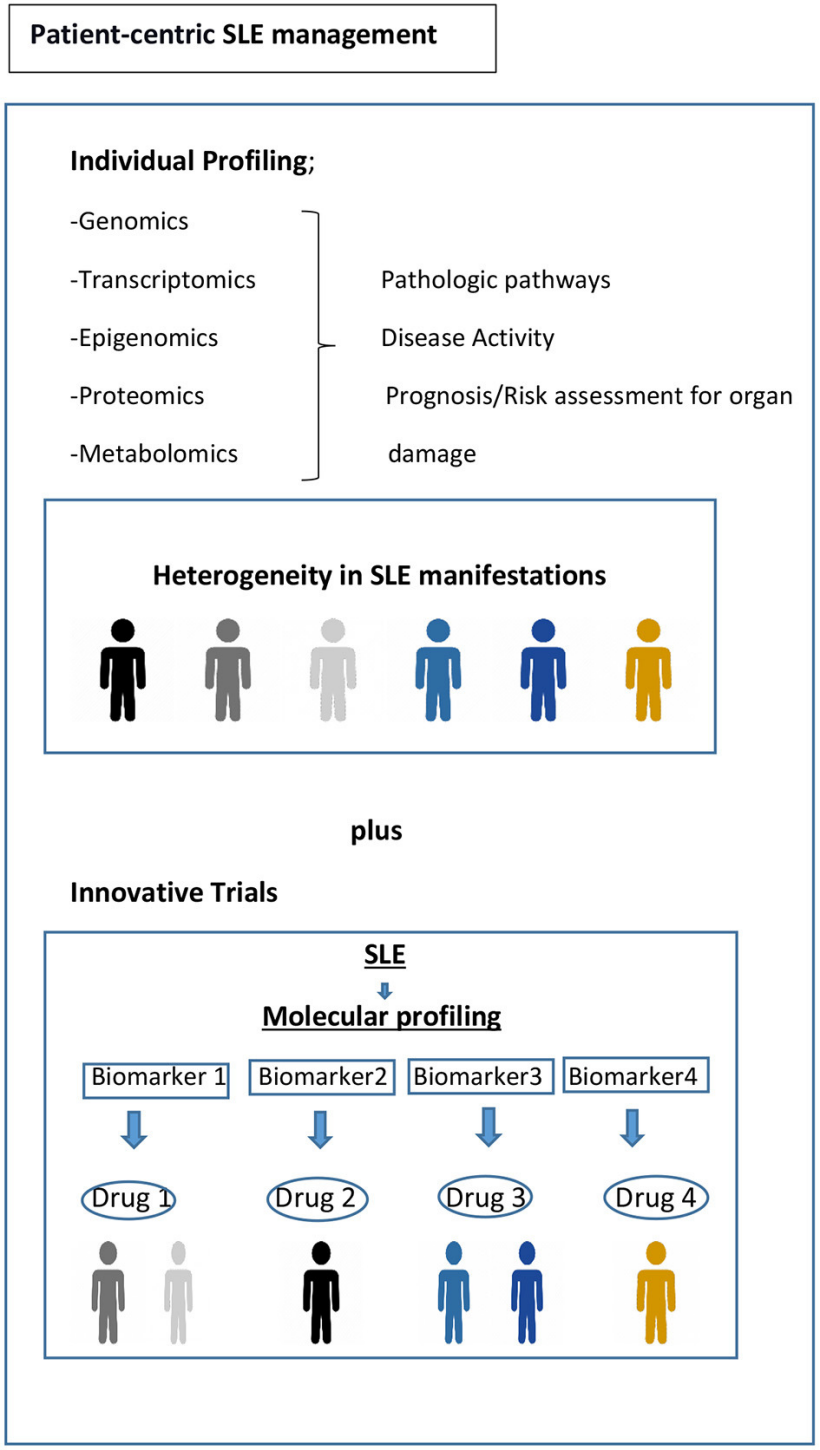
Toward precision medicine. Molecular profiling of patients at time of diagnosis and throughout their disease course would be helpful to assess the drivers of main pathophysiological pathways, to predict organ damage accrual and flares, and to offer optimum treatment and follow-up. Biomarker driven trial designs are proposed here. Adapted from REF (93).

Current randomized controlled trial (RCT) strategies assume that the target population is homogenous, for example, with respect to disease activity or serology. However, substantial biologic heterogeneity and its potential link to diverse clinical phenotypes in SLE have already been underscored in several studies (45, 63). Stratifying patients based on underlying molecular pathology is a tantalizing prospect using innovative patient-centric targeted treatment designs that may enhance response rates, as already observed in oncology (Figure 1) (93). Recent trials with anifrolumab (94), which blocks type 1 interferon, and ongoing trials for Atacicept (blocks BAFF and APRIL) (95), demonstrate the possibility of success using approaches that are more precise in the treatment of SLE. Earlier data suggested that the IGS would facilitate better discernment of the SLE patients who might respond to anifrolumab and possibly serve as a biomarker (96). The results of the TULIP-2 trial did show a greater treatment effect in patients with a high IGS, but not greatly different from those with a low IGS. It is unclear whether this result is related to assay performance, timing, or something else; it certainly needs further exploration. The incorporation of molecular phenotypes into clinical trials in SLE has also been tested using the results of the ADDRESS II study of atacicept. The investigators carried out a cell deconvolution algorithm in the baseline gene expression profiles to confirm the differential treatment effect observed in the ADDRESS II study (97). In this exploratory study, they showed that patients with high B cell or plasmablast gene expression, especially within the high disease activity group, demonstrated a better atacicept treatment response, which is relevant to the proposed mechanism of action of the drug. Nevertheless, the phase 2 trial failed to meet its primary clinical endpoint.

Other important considerations are the variations in treatment response between ethnicities that have been shown in different studies (11, 18). For example, African ancestry (AA) patients with LN may have a more favorable response to rituximab (15), which is compatible with the gene signature seen in AA patients that is characterized by a perturbed B cell axis (12). Although rituximab (19) failed to show a significant effect compared to standard of care in patients with LN in the LUNAR trial, there is consensus that in some patients, B cell deletion by targeting CD20 is beneficial (98, 99). In practice, many physicians use rituximab as a last resort for those who failed to respond to the current SOC (86). A recent study also pointed out the importance of taking into account genetic differences across ancestries that may result in differences in response to drug targets (100). By utilizing expression quantitative trait loci (eQTL) mapping and a comprehensive systems biology approach for predicting SLE-associated pathways, the investigators identified both common and ancestry specific associations. Whereas, the pathways related to innate immune and myeloid cell functions were enriched in patients of European ancestry (EA), the pathways associated with aberrant B cell activity along with the ER stress and metabolic dysfunction were enriched in patients of AA.

### Drug Repurposing in SLE

Drug repurposing analyses can identify a new therapeutic use of a drug based on the ability to revert a pathological gene expression signature in a condition that is not its primary indication in clinical practice (101, 102). As a robust alternative to *de novo* drug discovery, drug repurposing analyses have the advantage of employing many data-driven strategies that integrate multiple sources of data. Given the paucity of licensed therapeutic agents for SLE, these have also been performed in SLE (103, 104). The comparison of transcriptomic data from longitudinally followed SLE patients with drug-induced gene signatures from the widely used analytical tool, Connectivity Map Linked User Environment (CLUE) database, revealed differences in drug-induced gene-expression connectivity scores based on the patient subset enriched for neutrophils and lymphocytes (104). However, further clinical studies are required to determine whether this type of sub-setting of patients can improve the treatment or indicate novel therapies.

## Conclusion

The current landscape of research in genetic(s)/omic(s) presents an opportunity for the integration of molecular phenotype and clinical phenotype to address unmet needs in SLE. Recent advances in new technologies and analytical approaches have facilitated the discovery of molecular pathways, potential biomarker candidates, and drug repurposing efforts that may improve classification and treatment of patients with SLE. However, shifting from the current paradigms in patient care and drug development will require the demonstration of the clinical utility of these advances using innovative clinical trials. Despite the challenges, the future for patients with SLE seems propitious. Patient-centric precision medicine has the potential to offer the proper drug(s) for a patient's condition determined by individual molecular phenotype, thereby targeting disease pathophysiology and treatment response. However, achieving this will require consensus within academia, physicians, industry, patient organizations, and regulatory agencies.

## Author Contributions

SY and PL wrote the manuscript. All authors read, provided critical review, and accepted the final version of the manuscript.

## Conflict of Interest

The authors declare that the research was conducted in the absence of any commercial or financial relationships that could be construed as a potential conflict of interest.
