# Supplementary material for: Current Status of the Evaluation and Management of Lupus Patients and Future Prospects
Source: Front Med (Lausanne). 2021 May 28;8:682544. doi: 10.3389/fmed.2021.682544 (PMC8193052; doi:10.3389/fmed.2021.682544)
Supplement: Supplementary file 1 [file Table_1.DOCX]

**Table S1. Comparative sensitivity and specificity of systemic lupus erythematosus (SLE) classification criteria across disease**

**duration**

|  | Sensitivity |  |  | Specificity |  |  |
| --- | --- | --- | --- | --- | --- | --- |
| Disease duration | **ACR1982/1997**  **Criteria** | **95%CI**  **SLICC 2012 Criteria** | **EULAR/ACR 2019 Criteria** | **ACR1982/1997**  **Criteria** | **95%CI**  **SLICC 2012 Criteria** | **EULAR/ACR 2019 Criteria** |
| <1 year | 0.56  0.21-0.86 | 0.89  0.52-0.99 | 0.89  0.52-1.00 | 0.92  0.74-0.99 | 0.92  0.74-0.99 | 0.92  0.74-0.99 |
| 1 to <3years | 0.81  0.72-0.88 | 0.98  0.93-1.00 | 0.97  0.92-0.99 | 0.95  0.92-0.99 | 0.88  0.80-0.94 | 0.96  0.90-0.99 |
| 3 to <5 years | 0.81  0.70-0.90 | 0.91  0.82-0.97 | 0.96  0.88-0.99 | 0.94  0.87-0.98 | 0.89  0.80-0.94 | 0.99  0.94-1.00 |
| ≥ 5 years | 0.84  0.80-0.87 | 0.97  0.96-0.99 | 0.96  0.94-0.98 | 0.93  0.90-0.95 | 0.81  0.76-0.85 | 0.93  0.89-0.95 |

ACR, American College of Rheumatology; EULAR, European League Against Rheumatism; SLICC, Systemic Lupus International Collaborating Clinics. Adapted from REF^38^
